# *In Vitro* Antimicrobial Activity and Effect on Biofilm Production of a White Grape Juice (*Vitis vinifera*) Extract

**DOI:** 10.1155/2015/856243

**Published:** 2015-12-07

**Authors:** Angela Filocamo, Carlo Bisignano, Giuseppina Mandalari, Michele Navarra

**Affiliations:** Department of Chemical, Biological, Pharmaceutical and Environmental Science, University of Messina, Viale Annunziata, 98168 Messina, Italy

## Abstract

*Background*. The aim of the present study was to evaluate the antimicrobial effect of a white grape juice extract (WGJe) against a range of Gram-positive and Gram-negative bacteria, yeasts, and the fungus* Aspergillus niger*. WGJe was also tested on the production of bacterial biofilms* in vitro*.* Results*. WGJe inhibited* in vitro* most Gram-positive bacteria tested,* Staphylococcus aureus* ATCC 6538P being the most sensitive strain (MIC values of 3.9 *μ*g/mL). The effect was bactericidal at the concentration of 500 *μ*g/mL. Amongst the Gram-negative bacteria,* Escherichia coli* was the only susceptible strain (MIC and MBC of 2000 *μ*g/mL). No effect on the growth of* Candida* sp. and the fungus* Aspergillus niger* was detected (MIC values > 2000 *μ*g/mL). WGJe inhibited the biofilms formation of* E. coli* and* Pseudomonas aeruginosa* with a dose-dependent effect.* Conclusions*. WGJe exerted both bacteriostatic and bactericidal activity* in vitro*. The presented results could be used to develop novel strategies for the treatment of skin infections and against potential respiratory pathogens.

## 1. Introduction

A wide variety of compounds have been identified in grapes, most of which with health promoting properties. A number of studies have shown that beneficial effects of grape and grape products consumption are related to the presence of polyphenols, mainly flavonoids and phenolic acids, with antioxidant, anti-inflammatory, antimicrobial, antiviral, and cancer preventive properties [1–4]. Flavonoids, stilbene, and proanthocyanidins are considered the most abundant class of bioactive compounds in grapes. It has also been demonstrated that grape juices, both white and purple, represent an important source of minerals, which would help explain the antioxidant and antimutagenic properties of grapes [5]. We have recently shown that an extract from white grape juice could have a beneficial effect on radiocontrast medium toxicity in human renal proximal tubular cells [6] and exert neuroprotective effect in a mouse model of experimental autoimmune encephalomyelitis [7]. Due to the increased antibiotic resistance, the antimicrobial properties of natural compounds have been gaining attention: we have previously reported the antibacterial activity of polyphenols-rich natural products, including almonds [8, 9], pistachios [10],* Citrus* plants [11],* Vitis vinifera* L. [12],* Olea europaea* L. [13],* Citrus bergamia* essential oil [14], and juice [15]. In particular, the identification of novel compounds with bactericidal rather than bacteriostatic effect has attracted interest in recent years. Inhibition and eradication of Gram-positive and Gram-negative biofilms have also been proven to be rather difficult with conventional antibiotics, with a need for novel antimicrobials able to treat biofilm-related infections. Yadav et al. [16] have recently demonstrated that eugenol was effective against methicillin-resistant and methicillin-sensitive* Staphylococcus aureus* clinical strain biofilms. The effects of cranberry extracts have also been evaluated on the growth and biofilm production of* Escherichia coli* and* Staphylococcus* sp. [17].

In the present study we evaluated the antimicrobial effect of a polyphenols-rich white grape juice extract against Gram-positive and Gram-negative bacteria, yeasts, and the fungus* Aspergillus niger*. Furthermore, the same extract was tested on the production of bacterial biofilms* in vitro*.

## 2. Materials and Methods

### 2.1. Grape Juice Extract

A white grape (*Vitis vinifera*) juice extract (WGJe) was kindly provided by “Bono & Ditta” (Campobello di Mazzara, Trapani, Italy). The extract derived from a mixture of white grapes juice from* Vitis vinifera *var. Catarratto,* Vitis vinifera* var. Grillo, and* Vitis vinifera* var. Insolia. WGJe was produced in its liquid form by passing the must-mute columns equipped with adsorbent resins, known to retain polyphenols. Molecules were then eluted with 4% NaOH and immediately passed through cationic resins, thus obtaining an acidic form. Products were collected, filtered, and then sprayed to obtain a dry powder kept at −20°C until further use [6, 7]. WGJe chemical composition, evaluated by UPLC/QqQ-MS/MS, has been previously reported [6, 7]. The major polyphenols identified in WGJe were quercetin-3-glucuronide, procyanidin B1, quercetin-3-glucoside, catechin, and* t*-coutaric acid. Minor identified compounds included a number of glucosides, such as kaempferol-3-glucuronide and kaempferol-3-glucoside, isorhamnetin-3-glucoside, and quercetin-3-glucoside-arabinoside. Phenolic acids, including vanillic acid, ellagic acid, ferulic acid, chlorogenic acid, caffeic acid, and* p*-coumaric acid, were also identified.

### 2.2. Microbial Strains and Culture Conditions

The following strains from the University of Messina's in-house culture collection (Messina, Italy) were used for the antimicrobial testing:* Staphylococcus aureus* ATCC 6538P,* Staphylococcus aureus* MRSA ATCC 43300,* Staphylococcus epidermidis* ATCC 49134,* Staphylococcus epidermidis* ATCC 35984,* Staphylococcus epidermidis* ATCC 12228,* Streptococcus pneumoniae* ATCC 6003,* Streptococcus pyogenes* ATCC 19615,* Streptococcus mutans* ATCC 35668,* Listeria monocytogenes* ATCC 7644,* Listeria monocytogenes* ATCC 1392,* Enterococcus hirae* ATCC 10541,* Moraxella catarrhalis* ATCC 8176,* Bacillus subtilis* ATCC 8176,* Enterococcus durans* (wild-type strain),* Escherichia coli* ATCC 25922,* Klebsiella pneumoniae* (wild-type strain),* Pseudomonas aeruginosa* ATCC 27853,* Pseudomonas aeruginosa* ATCC 9027,* Pseudomonas aeruginosa* ATCC 15442,* Proteus mirabilis* (wild-type strain),* Serratia marcescens* (wild-type strain),* Salmonella typhi* ATCC 13311,* Candida albicans* ATCC 10231,* Candida parapsilosis* ATCC 29947, and the fungus* Aspergillus niger* ATCC 16404.

Bacteria were grown in Mueller-Hinton Broth (MHB, Oxoid, CM0405) at 37°C (24 h), whereas yeasts were cultured in Sabouraud Liquid Medium (SLM, Oxoid, CM0147) at 30°C (48 h). For solid media, 1.5% (w/v) agar (Difco) was added.* Aspergillus niger* was grown in Sabouraud Dextrose Agar at 30°C for 7 days as previously reported [10].

### 2.3. Susceptibility Studies

For the susceptibility studies, WGJe was dissolved in sterile PBS at the concentration of 4 mg/mL; the pH of this solution was 7.10. The minimum inhibitory concentration (MIC), the minimum bactericidal concentration (MBC), and the minimum fungicidal concentration (MFC) of WGJe were determined by the broth microdilution method, according to CLSI [18]. Briefly, twofold serial dilutions of WGJe were added to MHB and inoculated into 96-microtiter plates with a final inoculum of approximately 5 × 10^5^ CFU mL^−1^. The tested concentrations ranged from 2000 to 3.9 *μ*g mL^−1^, and no significant changes of the pH of the growth medium were detected after addition of WGJe. The MIC was considered as the lowest concentration, which completely inhibited bacterial growth after 20 h.

The MBCs were determined by seeding 20 *μ*L from all clear MIC wells onto Mueller-Hinton agar (MHA, Oxoid) plates. The MBC was defined as the lowest extract concentration that killed 99.9% of the final inocula after 24 h incubation.

### 2.4. Effect on Biofilm Formation

The effect of different concentrations of WGJe on biofilm-forming ability was tested on polystyrene flat-bottomed microtiter plates as described by Nostro et al. [19].

Twofold serial dilutions of WGJe ranging from 500 to 62.5 *μ*g mL^−1^ were used for* E. coli* ATCC 25922 and* P. aeruginosa* ATCC 9027.

Cultures were grown overnight and suspensions adjusted to 10^6^ CFUmL^−1^. Aliquots of 100 mL were dispensed into each well of a sterile flat-bottomed 96-well polystyrene microtiter plates (Corning Inc., Corning, NY) in the presence of WGJe.

Planktonic growth was determined by spectrophotometric values (OD_492_ nm) after 24 h. The medium was then aspirated and the wells, rinsed twice with phosphate-buffered saline (PBS), were fixed by drying for 2 h. Once the wells were fully dry, 200 mL of 0.1% safranin was added for 2 min. The content of the wells was then aspirated, and, after rinsing with water, 200 mL of 30% acetic acid (v/v) was added. OD_492_ was measured by spectrophotometry using an ELISA reader.

Biofilm controls consisting of medium alone, medium plus strains (without WGJe), and medium plus WGJe (without strain) were included.

Each assay was performed in duplicate and repeated at least three times.

## 3. Results

### 3.1. Minimum Inhibitory Concentrations

The MIC and MBC values of WGJe against the strains tested are shown in Table 1. WGJe was active against all the Gram-positive strains included in the study (MIC values between 3.9 and 1000 *μ*g mL^−1^), whereas no activity was found against the Gram-negative bacteria, with the exception of* E. coli* (MIC of 2000 *μ*g mL^−1^), the yeasts, and* A. niger* at the concentrations tested.

Amongst the Gram-positive bacteria,* S. aureus* ATCC 6538P showed the highest sensitivity, with MIC and MBC values equal to 3.9 and 500 *μ*g mL^−1^, respectively.

Similarly, a good growth inhibitory activity was found against* S. epidermidis* and* M. catarrhalis*, with MIC values of 15.62 *μ*g mL^−1^ and MBC values of 2000 *μ*g mL^−1^. WGJe (125 *μ*g mL^−1^) has also been able to inhibit the growth of* L. monocytogenes*,* S. mutans*,* S. pyogenes*, and* S. pneumonia*. MBC values of 1000 *μ*g mL^−1^ for* S. pyogenes* and* S. pneumonia* and 2000 *μ*g mL^−1^ for* S. mutans* were detected, whereas no bactericidal activity was found against* L. monocytogenes* at the concentrations tested.

MIC values of 250, 500, and 1000 *μ*g mL^−1^ were also found against* B. subtilis*,* E. durans*, and* E. hirae*, respectively. A MBC value corresponding to the maximum concentration tested was detected for* B. subtilis*, whereas the same concentration showed bacteriostatic activity on* E. durans* and* E. hirae*.

Amongst the Gram-negative bacteria, a slight inhibitory activity was found against* E. coli* for which the value of MIC found, however, corresponds to the highest concentration used. No activity was observed against other Gram-negative strains.

### 3.2. Effect on Biofilm Formation

Table 2 shows the effect of WGJe on the biofilm formation of* E. coli* and* P. aeruginosa*. Since the MIC value was relatively high for* E. coli* and no inhibition was observed against* P. aeruginosa* (Table 1), the WGJe concentrations used ranged between 500 and 62.5 *μ*g mL^−1^.

The results demonstrated that WGJe (500 and 250 *μ*g mL^−1^) produced a reduction on biofilm formation of 42.49% and 23.79% for* E. coli* and 30.47% and 23.88% for* P. aeruginosa*, respectively.

## 4. Discussion

The present study has demonstrated that WGJe was effective against a range of Gram-positive bacteria. Although WGJe did not affect the growth of any Gram-negative bacteria tested, with the exception of* E. coli*, a reduction on biofilm formation of both* E. coli* and* P. aeruginosa* was detected. A number of studies have previously investigated the antimicrobial effect of grape, wine, and their byproducts [20, 21]. Red wine has been shown to prevent damage to the gastric mucosa induced by* Helicobacter pylori*, possibly through inhibition of the vacA gene [22]. Jayaprakasha et al. [23] have demonstrated that grape seed extracts have antimicrobial potential, Gram-positive bacteria being more sensitive than Gram-negative bacteria. Our study has also demonstrated that Gram-positive strains were more susceptible to WGJe compared with Gram-negative bacteria, with a bactericidal effect observed on all Gram-positive strains tested, with the exception of* E. durans*,* E. hirae*,* Listeria* spp., and* S. epidermidis* ATCC 49134. A strong inhibitory effect against* Listeria monocytogenes* has been found by grape juice and grape extracts derived from* Vitis vinifera* variety “Ribier” [24]. Sanhueza et al. [25] have recently reported an antibacterial effect of grape pomace extracts mainly against* S. aureus* and* E. coli*: the activity was directly related to the polar phenolic content. Grape seed extracts obtained from wine and table cultivars of* Vitis vinifera* L. were found to be active against* Candida albicans* sp. and their activity was related to the presence of polymeric flavan-3-ols [26].

The antibacterial activity of polyphenols has been recently reviewed [27]. In agreement with our study, several wine phenolic acids, mainly gallic acid and ethyl gallate, were able to inhibit the growth of respiratory pathogenic bacteria and potential respiratory pathogens including* P. aeruginosa*,* S. aureus*,* M. catarrhalis*, and* E. faecalis* [28]. However, unlike the report by Cueva et al. [28], we found Gram-positive bacteria were more sensitive to WGJe compared with the Gram-negative tested strains. Some phenolic acids, such as cinnamic acid, ferulic acid,* p*-coumaric acid, and caffeic acid, were also found to be active against* Listeria* spp. [29]. Chlorogenic acid extracted from blueberry fruit was able to inhibit 46% and 42% of* S. epidermidis* and* P. aeruginosa* biofilm formation, respectively [30]. We believe that the phenolic acids present in WGJe played an important role in reducing biofilm formation of* E. coli* and* P. aeruginosa*, in a dose-dependent way.

## 5. Conclusions

The results of the present study demonstrated that WGJe was effective against a range of Gram-positive bacteria, including potential respiratory pathogens, and* E. coli* amongst Gram-negative strains. The exerted activity was both bacteriostatic and bactericidal. Furthermore, WGJe was able to inhibit the biofilm formation of* E. coli* and* P. aeruginosa in vitro*.

## Figures and Tables

**Table 1:** MICs and MBCs of WGJe (expressed as *μ*g/mL) against Gram-positive bacteria, Gram-negative bacteria, yeasts, and the fungus *A. niger*.

	MIC	MBC
Gram+		
*M. catarrhalis* ATCC 8176	15.62	2000
*B. subtilis* ATCC 6633	250	2000
*E. durans* (wild-type strain)	500	>2000
*E. hirae* ATCC 10541	1000	>2000
*L. monocytogenes* ATCC 7466	125	>2000
*L. monocytogenes* ATCC 13932	250	>2000
*S. aureus* ATCC 6538P	3.9	500
*S. aureus* ATCCC 43300	62.5	500
*S. epidermidis* ATCC 49134	15.62	>2000
*S. epidermidis* ATCC 12228	31.25	2000
*S. epidermidis* ATCC 35984	15.62	2000
*S. mutans* ATCC 35668	125	2000
*S. pyogenes* ATCC 19615	125	1000
*S. pneumoniae* ATCC 6003	125	1000
Gram−		
*E. coli* ATCC 25922	2000	2000
*K. pneumoniae* (wild-type strain)	>2000	>2000
*P. aeruginosa* ATCC 27853	>2000	>2000
*P. aeruginosa* ATCC 9027	>2000	>2000
*P. aeruginosa* ATCC15442	>2000	>2000
*P. mirabilis* (wild-type strain)	>2000	>2000
*S. marcescens* (wild-type strain)	>2000	>2000
*S. typhi* ATCC13311	>2000	>2000
Yeasts		
*C. albicans* ATCC 10231	>2000	>2000
*C. parapsilosis* ATCC 29947	>2000	>2000
Fungi		
*A. niger* ATCC 16404	>2000	>2000

MICs, minimal inhibitory concentrations; MBCs, minimal bactericidal concentrations; WGJe, white grape juice extract.

**Table 2:** Percentage biofilm reduction for twofold serial dilutions of WGJe ranging from 500 to 62.5 *µ*g mL^−1^ on *E. coli* ATCC 25922 and *P. aeruginosa* ATCC 9027.

	500	250	125	62.5
*E. coli *ATCC 10536	42.49 ± 0.75	23.79 ± 0.48	19.28 ± 0.33	10.55 ± 0.78
*P. aeruginosa *ATCC 9027	30.47 ± 0.27	23.88 ± 0.14	21.20 ± 0.19	17.38 ± 0.37

WGJe, white grape juice extract.
